# Bacterial biodiversity and optimization of pilot plant-based storage parameters of beet thick juice under Egyptian environmental conditions

**DOI:** 10.1038/s41598-025-99870-4

**Published:** 2025-05-16

**Authors:** Marwa Abdelhak, Osama Abdel-Hafeez Mohamed Al-Bedak, Mahmoud N. Abdelmoez, Adel Ahmed Abdellah, El-Sayed A. Abdel-Rahman, Mohamed M. Abd El-Wahab

**Affiliations:** 1 Department of Science and Technology of Sugar Industry, Faculty of Sugar and Integrated Industries Technology, Assiut University, Assiut, 71511 Egypt; 2 Assiut University Mycological Centre (AUMC), Assiut University, Assiut, 71511 Egypt; 3 ERU Science & Innovation Center of Excellence, Egyptian Russian University, Badr city, Cairo, 11829 Egypt; 4 Department of Mechanical Power Engineering, Faculty of Engineering, Assiut University, Assiut, 71511 Egypt; 5 Institute for Life and Medical Sciences, Kyoto University, Kyoto, Japan; 6 Alexandria Sugar Company (Savola Foods), Burg El Arab 21934, Alexandria, Egypt; 7 Department of Food Science and Technology, Faculty of Agriculture, Assiut University, Assiut, 71511 Egypt; 8 Department of Chemistry, Faculty of Science, Assiut University, Assiut, Egypt

**Keywords:** Antibacterial, Biocides, Hop-β-acids, Storage, Sugar beet, Thick juice, 16SrRNA, Biotechnology, Microbiology, Molecular biology

## Abstract

In hot-weather regions such as Egypt, where maintaining lower storage temperatures is both economically unfeasible and a significantly difficult, this study was directed towards optimizing the storage of beet thick juice in order to enhance white sugar yield and reduce the impact of climate change. In this study, a pilot plant consisting of 12 storage cylinders was used to maintain thick beet juice with total soluble solids (ºBrix) of 67, 68, and 69 at 15, 25, and 35 ºC, respectively. Betastab^®^ XL (Hop ß-acids) and KEBOCID 310 (sodium dimethyldithiocarbamate) were employed as biocides at 40 ppm. Surface sealing using 25.0% NaOH along with air removal was also applied. The relationships between bacterial count (CFUs), pH, lactic acid (LA) concentration, and reducing sugars (RS) content were evaluated. CFUs increased gradually with the length of the storage period in all tanks, reaching its peak (350 CFU/mL) in the control tank at 35 °C. LA and RS values were determined in all tanks and occurred at very low concentrations. pH exhibited a stable behavior with the exception of the control tank at 35 ºC that displayed degradation correlated to a pH drop to 7.32. Species of bacteria associated with the stored thick juice were isolated and identified by sequencing the 16 S rRNA. These included *Bacillus cereus*, *B. licheniformis*, *B. paralicheniformis*, *B. subtilis*, *Bordetella muralis*, *Brevibacillus agri*, *Pseudomonas juntendi*, and *Stenotrophomonas geniculata*. Antibacterial activity of both biocides at three concentrations—20, 40, and 60 ppm—was investigated against the species isolated. Hop ß-acid concentrations showed significant effects on the investigated bacteria with the exception of *B. muralis* and *P. juntendi*. All bacteria, with the exception of *B. licheniformis* AUMC B-550, were significantly affected by KEBOCID 310 concentrations. However, *S. geniculata* responded negatively to 20 ppm of KEBOCID 310. For the investigated strains, the effects of KEBOCID 310 at 40 and 60 ppm were almost identical. Along with better control over juice solids content and storage temperature, identifying the source and type of the bacterial infection can assist with managing the process and reduce thick juice deterioration. Sugar industry designers could potentially benefit from this study’s elucidation of the ideal circumstances for cost-effective storage in hot temperature zones.

**Supplementary Information:**

The online version contains supplementary material available at 10.1038/s41598-025-99870-4.

## Introduction

Since its introduction in the United States in 1960, numerous sugar companies have been using industrial thick juice storage on a regular basis. The thick juice is extracted by the sugar factories using a technological plan for long-term storage, and the thick juice is subsequently processed in the off-season. This strategy helps sugar factories to increase productivity without incurring high costs^1–4^. Beet thick juice is the intermediate product after evaporation with a total soluble solids content of about 69 °Brix (°Bx) and a slightly alkaline pH of 9.0^5^. The potential for microbes to infect the thick juice is a disadvantage of the storage approach, even with optimal preservation procedures. Thick juice deterioration as a result of microbial contamination has been observed in industrial practices^6,7^. The most evident signs of deterioration are a pH drop from 9 to 5–7 and a usual rise in the concentration of reducing sugars, which increases sugar loss.

It is uncertain which particular bacteria are responsible for the degradation of the thick juice^8,9^. Sargent, et al.^8^ linked the pH drop to mesophilic bacteria, while Willems, et al.^9^ suggested a connection between thick juice degradation and fastidious bacteria (FB), that grow on growth media enriched with blood instead of conventional bacterial plating media. Many studies have examined the microbiota of sugar beets and the extraction juice^10–12^; however, very few investigations have documented the presence and growth of bacteria in the highly concentrated thick juice^9,13^. In line with the concept of chemical-free manufacturing, natural biocides have been increasingly investigated for their antibacterial properties during thick juice preservation^14^. Hop (*Humulus lupulus* L.) products were initially successfully used in the sugar industry in 1994 to inhibit microorganisms during beet juice extraction^15^. These naturally-occurring hop biocides are harmless for both humans and animals, and they even have a crucial role in beer industry^16^.

Egypt does not use any approach that extends the manufacturing season beyond the main season since not enough research has been carried out to determine the suitability of thick juice preservation technologies for the country’s environment. Therefore, this study was directed towards evaluating the potential for various treatments to be used for long-term preservation of beet thick juice in countries with hot climates. It also identified bacterial biodiversity and examined the influence of biocides on bacterial activity after six months of storage. Three matrix tests were used to examine the influence of some chemical parameters—pH, lactic acid concentration, and reducing sugar content—on the stability of the beet thick juice during storage at 15, 25, and 35 °C under controlled conditions. A 10.0% aqueous alkaline solution of hop-ß-acids, KEBOCID 310, and 25.0% NaOH, as preservatives were employed.

## Results

### Isolation and molecular identification of bacteria from stored thick juice

**Fig. 1:**
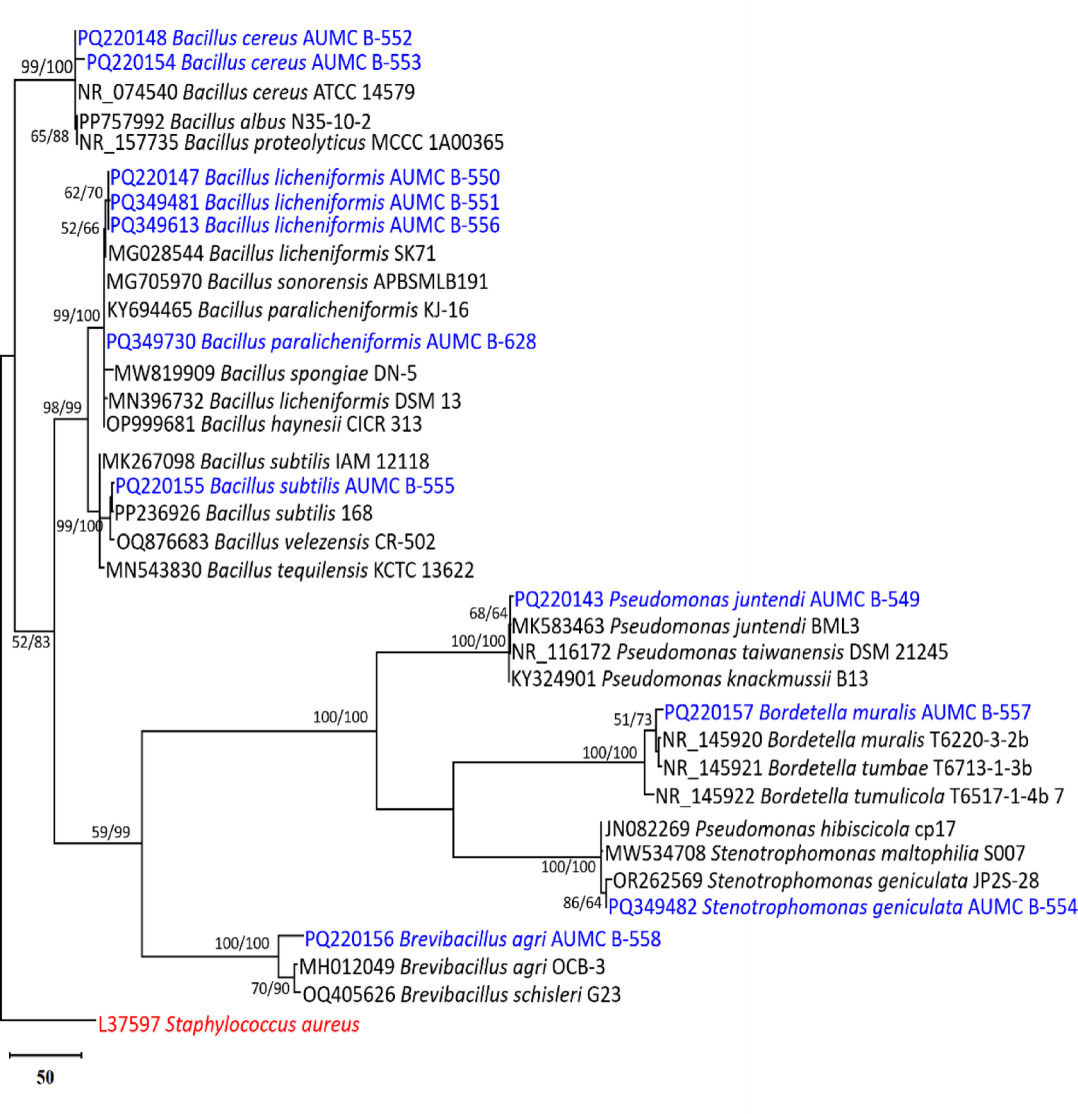
Maximum parsimonious phylogenetic tree generated from a heuristic search (1000 replications) of maximum likelihood/maximum parsimony analysis (ML/MP) of 16 S rRNA sequences of bacterial strains isolated in this study (in blue) compared to the most similar species in GenBank. Bootstrap support values of ML/MP ≥ 50% are referred near the respective nodes. The tree is rooted to *Staphylococcus aureus* (L37597) (in red).

The current study obtained 105 bacterial isolates. These were categorized into eleven groups based on their colony morphology, color, and Gram staining. Sequencing of the 16 S rRNA was used to identify one member of each group. Using phylogenetic analysis based on the 16 S rRNA dataset in comparison to the most closely related species published in GenBank, the taxonomic status of the 11 bacterial isolates used in this study was determined. The entire 16 S rRNA data set contained 36 species. In the maximum parsimony dataset, 1348 characters out of 1447 could align perfectly (no gaps, no N), 466 characters were classified as parsimony informative (34.6% of constant characters), and 489 were classified as variable characters (36.3% of constant characters). The evolutionary history was inferred by applying Maximum Likelihood and Maximum Parsimony analyses. Tamura-Nei using a discrete Gamma distribution (TN93 + G), was the best model to monitor the phylogenetic tree. The maximum parsimony analysis produced four trees, the most parsimonious one is shown in Fig. 1. The phylogenetic tree obtained displayed a tree length of 999, a log likelihood of -6587.56, a consistency index of 0.761210, a composite index of 0.720624, and a retention index of 0.946682.

## Phylogeny

Eight species of bacteria were identified through phylogenetic analysis. These were *Bacillus cereus* (2 strains), *Bacillus licheniformis* (3 strains), and *Bacillus paralicheniformis*, *Bacillus subtilis*, *Bordetella muralis*, *Brevibacillus agri*, *Pseudomonas juntendi*, *Stenotrophomonas geniculata* (one each). The MP tree revealed strong bootstrap support for most of the terminal clades and the tree backbone. Every strain was found within the clade that supported high bootstrap support values for ML/MP.

## Impact of storage time on the microbial and chemical parameters

### Impact on bacterial count

**Fig. 2:**
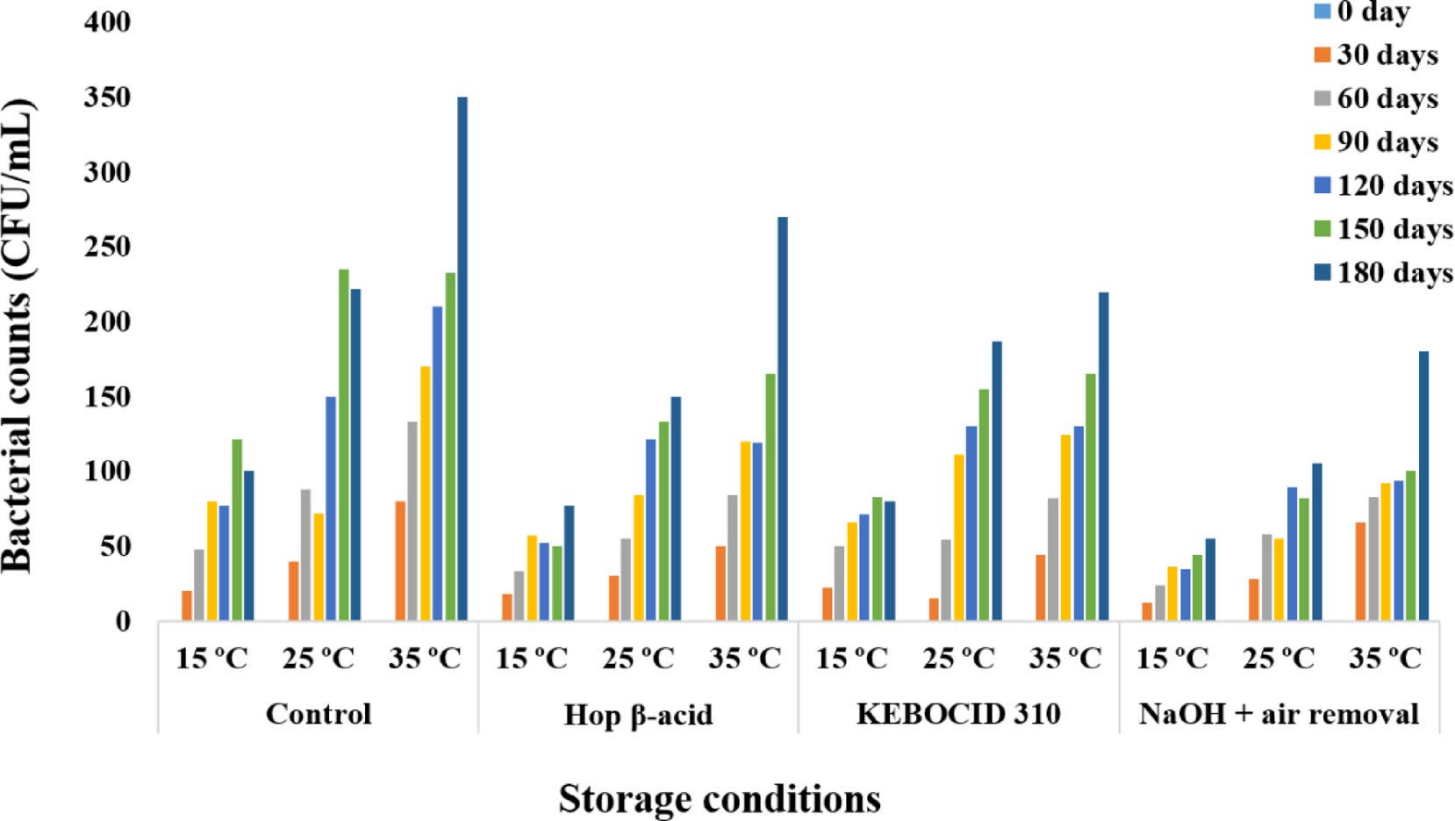
Trends in microbial counts for control and treated tanks at storage temperatures of 15, 25, and 35 ºC within the 180 day storage period of the beet thick juice.

Our findings validated the critical role that temperature plays during 180 days of thick juice storage. At 15 ºC, the CFUs grew slightly (121 CFU/mL), but they stayed within the safe limit for the control tank. CFUs in tanks treated with hop-ß-acid and KEBOCID 310 demonstrated 77 and 83 CFU/mL, respectively. The surface treatment with 25.0% NaOH and air removal resulted in 55 CFU/mL of the bacterial count. CFUs for the control tank increased with time at 25 ºC, reaching 235 CFU/mL by day 150. Furthermore, after the storage period, CFUs in tanks treated with hop-ß-acid and KEBOCID 310 grew to 150 and 187 CFU/mL, respectively. On day 180, the surface treatment had progressed to a bacterial count of 105 CFU/mL. At 35 ºC, upon reaching the end of the storage period for the control tank, the bacterial count rose to 350 CFU/mL. Moreover, CFUs in tanks treated with hop-ß-acid and KEBOCID 310 increased to 270 and 220 CFUs/mL, respectively, while in the surface treatment tanks it reached 180 CFUs/mL (Fig. 2).

## Impact on pH

**Fig. 3:**
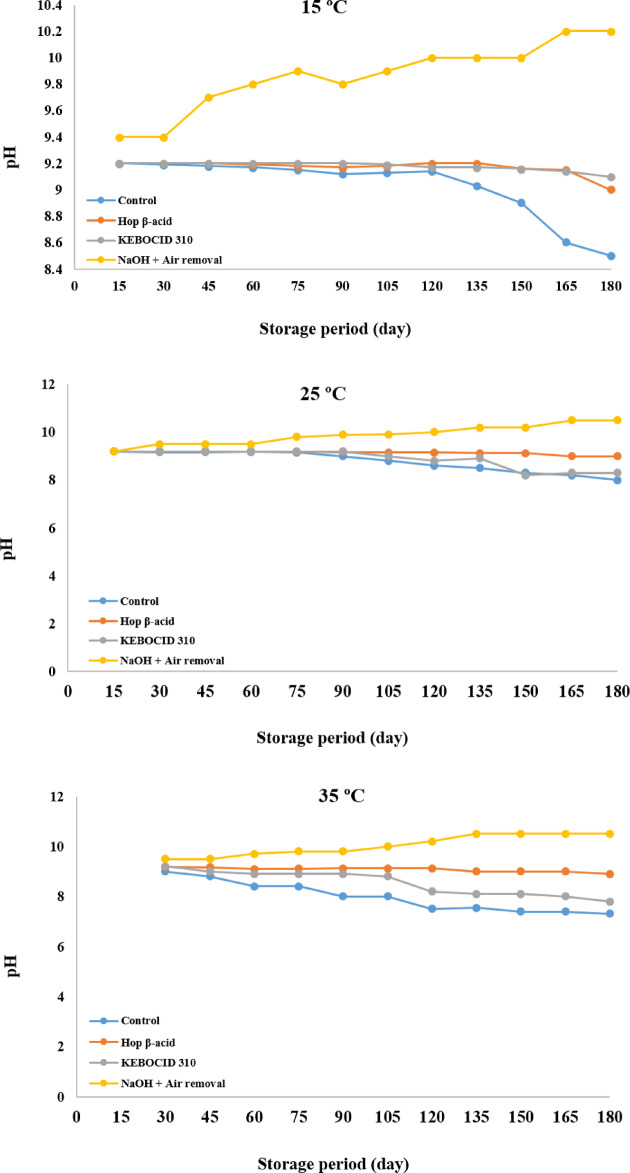
Effect of storage time of beet thick juice on pH at storage temperatures of 15, 25, and 35 ºC.

At 15 ºC, pH declined to 8.5 in the control tank, but in the other tanks that received Hop ß-acids and KEBOCID 310, it remained stable. However, in the tanks with the surface treatment, it increased to 10.2. At 25 ºC, pH of the control tank declined to 8.0, while in the other tanks that received hop-ß-acid and KEBOCID 310, it remained relatively constant. However, in tanks with surface treatment, it increased to 10.5. At 35 ºC, pH dropped sharply to 7.32 in the control tank by the day 180, while it declined to 8.9 and 7.8 in tanks with hop-ß-acid and KEBOCID 310. However, it increased to 10.5 in the tank with the surface treatment (Fig. 3).

### Impact on lactic acid concentration

At 15 ºC, in the control tank lactic acid was detected at the completion of the storage period (150–180 days) but at a very low concentration (50 ppm). It achieved its lowest concentration (50 ppm) at the completion of storage time in tanks with the surface treatment and biocides (50 and 80 ppm, respectively). At 25 ºC, at the end of storage, 120 ppm of lactic acid was detected in the control tank, as well as tanks treated with hop-ß-acid and KEBOCID 310. Lactic acid reached 80 ppm at the completion of storage while it was in the surface treatment tank. At 35 ºC, lactic acid was detected in the control tank with a concentration of 220 ppm at the end of the storage period. In tanks with hop-ß-acid and KEBOCID 310, it attained 120 ppm, and 80 ppm was determined for the surface treatment tank (Table 1).

## Impact on reducing sugars

At the end of the storage period, the reducing sugar content in the control tank had increased to 0.17% from an incredibly low concentration of 0.01%. The concentration (0.01–0.02%) remained constant in other tanks with surface treatment or with the Hop ß-acids and KEBOCID 310 addition. It was determined that at the end of the storage period, the reducing sugar content was declining and had reached 0.2% in the control tank. However, it attained only 0.15 and 0.12% in tanks treated with hop-ß-acid and KEBOCID 310, respectively. However, with the surface treatment tank, however, reducing sugars displayed a consistent and low concentration of 0.02%. Reducing sugars reached 0.5% at the conclusion of the storage time. However, it only reached 0.14 and 0.2% in tanks treated with hop-ß-acid and KEBOCID 310, respectively. While the surface treatment tank showed a low concentration of 0.12% (Table 2).

**Table 1:** Effect of storage time on beet thick juice on lactic acid concentration at the storage temperatures of 15, 25, and 35 ºC.

Storage time (day)	15 ºC				25 ºC				35 ºC			
	Control	Hop β-acid	KEBOCID 310	NaOH + Air removal	Control	Hop β-acid	KEBOCID 310	NaOH + Air removal	Control	Hop β-acid	KEBOCID 310	NaOH + Air removal
0	0	0	0	0	0	0	0	0	0	0	0	0
15	0	0	0	0	0	0	0	0	0	0	0	0
30	0	0	0	0	0	0	0	0	50	0	0	0
45	0	0	0	0	0	0	0	0	50	50	50	0
60	0	0	0	0	0	0	0	0	50	50	50	0
75	0	0	0	0	0	0	0	0	50	50	50	50
90	0	0	0	0	0	0	50	0	80	50	80	50
105	0	0	0	0	0	0	50	0	80	50	80	50
120	0	0	0	0	80	50	50	0	80	50	80	50
135	0	0	0	0	120	80	50	50	100	50	80	80
150	50	0	0	0	120	80	50	50	150	80	120	80
165	50	0	0	50	120	120	80	80	180	120	120	80
180	50	50	80	50	120	120	120	80	220	120	120	80

**Table 2:** Effect of storage time on beet thick juice on the reducing sugar content at the storage temperatures of 15, 25, and 35 ºC.

Storage time (day)	15 ºC				25 ºC				35 ºC			
	Control	Hop β-acid	KEBOCID 310	NaOH + Air removal	Control	Hop β-acid	KEBOCID 310	NaOH + Air removal	Control	Hop β-acid	KEBOCID 310	NaOH + Air removal
0	0.01	0.01	0.01	0.01	0.01	0.01	0.01	0.01	0.01	0.01	0.01	0.01
15	0.01	0.01	0.01	0.01	0.01	0.03	0.01	0.01	0.01	0.01	0.01	0.01
30	0.01	0.01	0.01	0.01	0.01	0.03	0.03	0.01	0.02	0.01	0.02	0.02
45	0.01	0.01	0.01	0.01	0.02	0.05	0.03	0.01	0.02	0.01	0.02	0.02
60	0.02	0.01	0.01	0.01	0.02	0.05	0.03	0.01	0.05	0.01	0.02	0.02
75	0.02	0.01	0.01	0.01	0.02	0.05	0.05	0.01	0.08	0.05	0.05	0.02
90	0.02	0.01	0.01	0.01	0.03	0.08	0.06	0.01	0.1	0.08	0.08	0.05
105	0.02	0.01	0.01	0.01	0.03	0.08	0.08	0.01	0.2	0.089	0.1	0.08
120	0.02	0.01	0.01	0.01	0.05	0.08	0.08	0.01	0.3	0.12	0.18	0.08
135	0.02	0.01	0.01	0.01	0.08	0.089	0.085	0.01	0.3	0.12	0.2	0.08
150	0.1	0.01	0.01	0.01	0.1	0.12	0.12	0.01	0.35	0.13	0.2	0.1
165	0.17	0.01	0.01	0.02	0.2	0.15	0.12	0.02	0.41	0.12	0.2	0.1
180	0.17	0.02	0.01	0.02	0.2	0.15	0.12	0.02	0.5	0.14	0.2	0.12

In vitro impact of biocides on the bacterial growth.

**Fig. 4:**
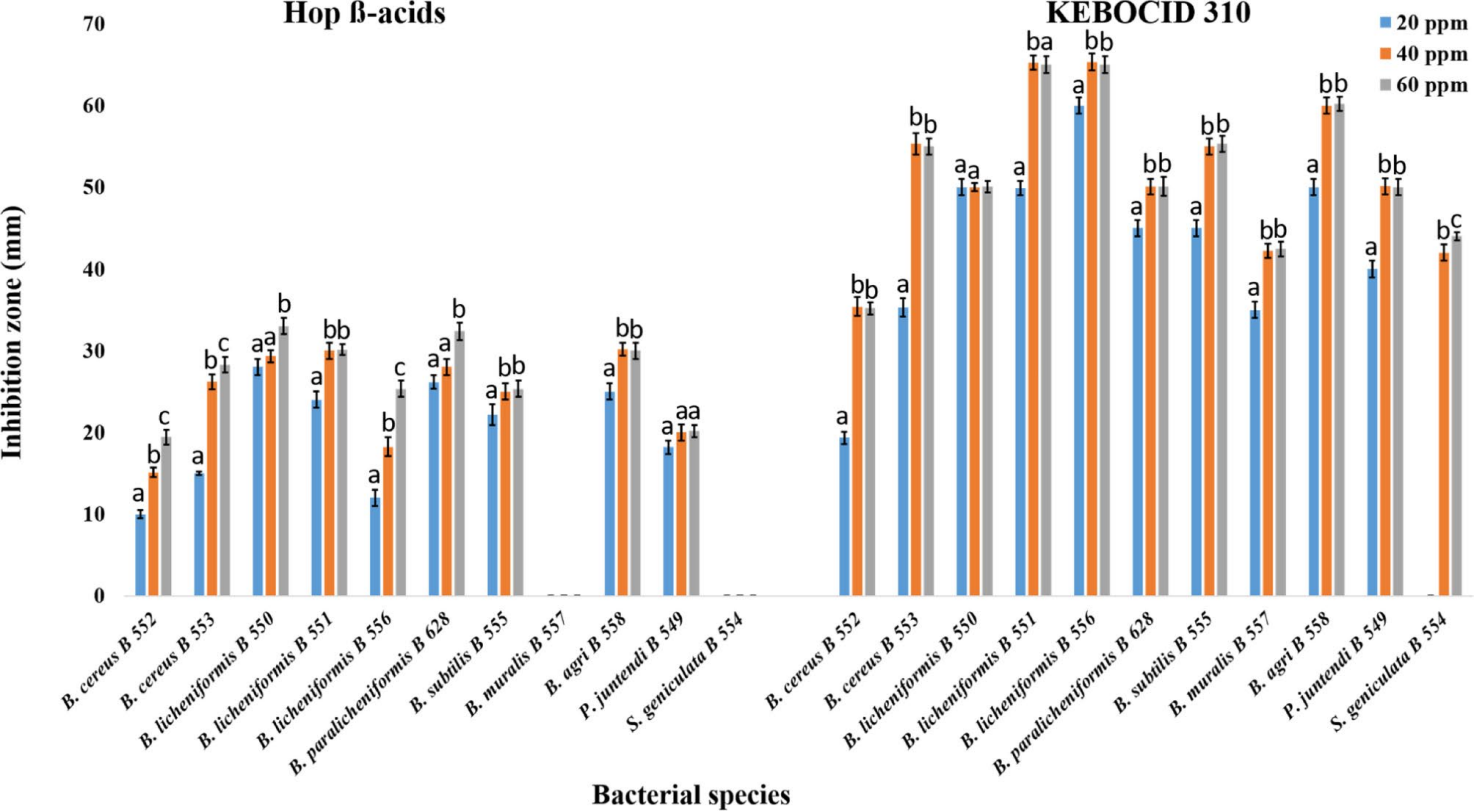
Effect of hop-ß-acid and KEBOCID 310 on the growth of the different strains of bacteria (expressed as inhibition in mm) isolated from stored thick juice (Mean values ± SD on graphs with different letters are significantly different; *p* < 0.01; *n* = 3).

At the selected concentrations of 20, 40, and 60 ppm, hop-ß-acid and KEBOCID 310 employed in this study demonstrated antibacterial effect at variable levels against all tested species. The inhibitory response of the studied bacterial strains increased significantly (*p* < 0.01) with increasing concentrations. With the exception of two species—*Bordetella muralis* AUMC B-557 and *Pseudomonas juntendi* AUMC B-549—all concentrations of hop-ß-acidhad a notable impact on the bacteria under study (F value = 7.432–263.044). On the other hand, KEBOCID 310 concentrations of 20, 40, and 60 ppm influenced all bacterial strains significantly (*p* < 0.01), with the exception of *B. licheniformis* AUMC B-550. (F values = 24.083–4444.8). *Stenotrophomonas geniculata* AUMC B-554 showed adverse effects from 20 ppm of KEBOCID 310. When applied at concentrations of 40 and 60 ppm, KEBOCID 310 produced almost identical effects on the investigated bacterial strains (Table 3; Fig. 4).

**Table 3:** Effect of hop-ß-acid and KEBOCID 310 biocides on growth of the different strains of bacteria (expressed as Inhibition in mm) isolated from stored thick juice (mean values ± sd with different letters are significantly different; *p* < 0.01; *n* = 3).

Bacterial strain	AUMC	Hop-ß-acids			F value	KEBOCID 310			F value
		20 ppm	40 ppm	60 ppm		20 ppm	40 ppm	60 ppm	
*Bacillus cereus*	B-552	10.0 ± 0.5^a^	15.1 ± 0.66^b^	19.43 ± 0.9^c^	135.27**	19.33 ± 0.76^a^	35.40 ± 1.15^b^	35.17 ± 0.76^b^	305.74**
*B. cereus*	B-553	15.0 ± 0.2^a^	26.2 ± 0.92^b^	28.27 ± 0.93^c^	263.044**	35.30 ± 1.1^a^	55.33 ± 1.33^b^	55.00 ± 1.0^b^	300.331**
*B. licheniformis*	B-550	28.0 ± 1.0^a^	29.33 ± 0.76^a^	33.00 ± 1.0^b^	23.355**	50.00 ± 1.0^a^	50.00 ± 0.5^a^	50.07 ± 0.7^a^	0.008
*B. licheniformis*	B-551	24.0 ± 1.0^a^	30.00 ± 1.0^b^	30.10 ± 0.66^b^	45.198**	49.90 ± 0.85^a^	65.23 ± 0.87^b^	65.00 ± 1.0^b^	278.648**
*B. licheniformis*	B-556	12.0 ± 1.0^a^	18.23 ± 1.17^b^	25.33 ± 1.0^c^	116.218**	60.00 ± 1.0^a^	65.33 ± 1.0^b^	65.00 ± 1.0^b^	26.054**
*B. paralicheniformis*	B-628	26.17 ± 0.86^a^	28.00 ± 1.0^a^	32.37 ± 1.1^b^	30.985**	45.00 ± 1.0^a^	50.10 ± 0.95^b^	50.10 ± 1.15^b^	24.1**
*B. subtilis*	B-555	22.17 ± 1.26^a^	25.00 ± 1.0^b^	25.33 ± 1.0^b^	7.432*	45.00 ± 1.0^a^	55.00 ± 1.0^b^	55.33 ± 1.0^b^	100.65**
*Bordetella muralis*	B-557	0.0	0.0	0.0	–	35.00 ± 1.0^a^	42.23 ± 0.87^b^	42.43 ± 0.93^b^	61.456**
*Brevibacillus agri*	B-558	25.0 ± 1.0^a^	30.17 ± 0.76^b^	30.00 ± 1.0^b^	30.032*	50.00 ± 1.0^a^	60.00 ± 1.0^b^	60.23 ± 0.87^b^	111.16**
*Pseudomonas juntendi*	B-549	18.2 ± 0.82^a^	20.00 ± 1.0^a^	20.17 ± 0.76^a^	4.75	40.0 ± 1.0^a^	50.13 ± 1.0^b^	50.00 ± 1.0^b^	100.9**
*Stenotrophomonas geniculata*	B-554	0.0	0.0	0.0	–	0 ± 0	42.00 ± 1.0^b^	44.00 ± 0.5^c^	4444.8**

* = significant difference; ** = highly significant difference.

## Discussion

Changes in the Egyptian sugar market have forced sugar producers to encourage strategies leading to the maximization of the sugar yield in order to manage the gap between sugar production and consumption. In this concern, efforts are being made to mimic the European approach for storing beet thick juice for processing at a later time following the main beet campaign. Shortening the beet campaign and safeguarding the crop from months of elevated heat in the Egyptian environment will lessen sugar loss. In addition, it will increase the end capacity of beet thick juice production without requiring additional factory construction or expenditure. Therefore, the objective of this study was to construct pilot-scale storage tanks with a low energy consumption, a cheap cost of cooling, and a workable design that simulate industry storage operations. It also was directed towards determining which storage techniques are most appropriate for application in industry.

Before filling, the tanks were thoroughly cleaned and sterilized as part of this study to reduce contamination on their inside surfaces. According to other studies^17^ and Pollach, et al.^18^ cleaning and sterilization are essential for preventing bacterial biofilms. Biofilms should be cleaned up as much as possible since they are more resistant to environmental stresses and are places where microorganisms can become established^19^. °Brix values were adjusted at 67, 68, and 69 in tanks at temperatures of 15, 25, and 35 °C, respectively in this investigation.

Many Egyptian sugar companies have the passion to follow the European strategy of preserving beet thick juice for processing up to several months after the campaign ends. Parameters such as ºBrix, pH, and temperature are known to be critical for controlling the stability of thick juice, as they directly impact microbial development. The temperature is the most important factor controlling the storage process of thick juice, and the selection of specific storage temperatures (15, 25, and 35 ºC) in this study was justified within the range of weather temperatures at the recommended months (from March to September) for industrial practice of thick juice storage at Alexandria Sugar Company in Egypt (Savola Foods, 30.819 N, 29.851 E). The weather conditions during the thick juice season recorded an average temperature of 23.4 ± 5.9 with a maximum temperature of 44.2 °C and a minimum of 7.7 °C (https://pvgis.com/pvgis-5-3). For the adjustment of total soluble solids (67, 68, and 69 ºBrix), it was calculated that each group of tanks depends upon its storage temperature and purity to maintain the thick juice stable at of supersaturation coefficient not more than 1 (saturated state) to prevent sucrose nucleation^6,7,9,20^. Juice deterioration has been studied under conditions that are nearly identical to ours (i.e., pH 8.6 to 9.6, dry solids concentration of 67 to 71 °Brix, and temperatures between 10 and 37 °C^8^. These optimized storage conditions were in accordance with those described by Asadi^20^. Considering that thick juice may spontaneously crystallize during the off-season while feeding the tanks, it was shown that °Brix values should be as high as feasible (68–69%) to avoid the formation of a low-dry-substance layer at the top of the tank^6^.

The initial pH of every tank in this study was adjusted to 9.2. In line with these results, Bohn^21^ and Moore^22^ realized that the rate of sucrose degradation diminishes with rising ºBrix and lowering storage temperature when thick juice was stored between pH 8.5 and pH 9.5. Reaffirming this, Gutknecht, et al.^23^ stated that a pH 9.0 was necessary for the long-term preservation of run-offs from the refining of raw cane sugar. Fiedler, et al.^24^ used experimental challenge tests to reach a similar conclusion. They demonstrated that pH 9.0 was preferable to pH 6.0, and a temperature of 5 °C was preferable to one of 15–20 °C or 30 °C for thick juice with a °Brix value lower than 67%. However, too high a pH causes colorization of the juice and an increase in reducing sugars resulting in sugar of a lesser quality^7,20^.

After 180 days of storage, the thick juice yielded 105 bacterial isolates for the current study, of which 92 isolates were of Gram positive and 13 of Gram negative bacteria. Eight species namely(*Bacillus cereus*, *B. licheniformis*, *B. paralicheniformis*, *B. subtilis*, *Bordetella muralis*, *Brevibacillus agri*, *Pseudomonas juntendi*, and *Stenotrophomonas geniculata*) were identified in this study using a 16 S rRNA sequencing approach. Our data indicate that determining the source and type of the bacterial contamination could improve process management and minimize thick juice degeneration in conjunction with improved control of juice solids content and storage temperature. Regarding this, only a small percentage of the several hundred strains of bacterial that were isolated from experimental cylinders and industrial storage tanks have been identified^7,9,25,26^. Cell morphology, Gram-stain, oxidase, and catalase reactions were among the fundamental tests used in bacterial identification. Currently, the best approach for identifying and recognizing several targets in a single experiment is DNA array technology^27–31^.

By rapidly and correctly identifying possible infectious agents, monitoring changes in microbial diversity during production enhances microbiological safety. It also facilitates developing a better understanding and management of the microbial processes involved in food processing^32–34^. As demanded by consumers, business, and governments, food quality and safety are supported by ecological research on microbial biodiversity as well as by accurate detection and trustworthy identification at the species level.

Three Gram-negative and five Gram-positive species of bacteria were found during the current study. According to the present findings, Gram-positive strains were primarily more prevalent than Gram-negative strains. Our findings are in line with those stated by Willems, et al.^9^, who concluded that the majority of the species of bacteria identified in thick juice were Lactobacilli, Actinobacteria, and Gram-positive Bacilli, with a tiny proportion of Gram-negative bacteria. The bacteria typically linked to thick juice spoilage are mostly Gram-positive^9,18,25,35,36^.

*Bacillus cereus*, *B. subtilis*, *Pseudomonas fragi*, and *Stenotrophomonas maltophilia* were previously isolated and identified from stored beet thick juice^9^. *Bacillus cereus*, *B. licheniformis*, *B. subtilis*, and *Pseudomonas* sp. also were isolated and identified from stored beet thick juice by Justé, et al.^7^. According to the available literature, *Bacillus paralicheniformis*, *Bordetella muralis*, *Brevibacillus agri*, *Pseudomonas juntendi*, and *Stenotrophomonas geniculata* were isolated from stored beet thick juice for the first time. Some isolated species of bacterial may be responsible for the change in pH and lactic acid generation. *Bacillus subtilis* was reported to produce lactic acid under this situation^37–39^. There are dangers of contamination when *B. cereus*–lactic acid-producing bacteria–are present in environments with high levels of sugar such those in the sugar industry. Their enzymatic properties and durability can cause sugar products to deteriorate and degrade^40,41^. *Bacillus licheniformis* has been shown in numerous studies to produce lactic acid^42–44^. These bacteria are endospore-forming, meaning they may withstand processing conditions and contaminate juice.

Only one species—the Gram-positive *B. paralicheniformis*—was predominantly isolated from all tanks during the 150–180–day storage period in the current study. This suggests that the strain is crucial to the deterioration of the beet thick juice in the control tank at 35 ºC. In this specific context, *Tetragenococcus halophilus* has been found to be the principal species of bacteria responsible for the degradation of beet thick juice^7^. Considering the lack of research on the isolation and identification of bacteria from stored thick juice, the comparison appears to be restricted.

Our findings demonstrated the impact of temperature on the microbiological development of the stored beet thick juice. For example, at storage temperatures of 15, 25, and 35 ºC, CFUs increased progressively in all tanks. At 35 ºC, CFUs showed the highest count (350 CFU/mL) in the control tank over 15 and 25 ºC (121 and 235 CFU/mL, respectively) by day 150 of storage. Despite this, the treated tanks at 35 ºC remained within a safe range that prevented juice from deteriorating. This could be explained by the use of biocides (hop-ß-acid and KEBOCID 310) in the storage experiment. Also, it might be attributed to the belief that bacterial growth became more rapid when the temperature reached the range that is ideal for bacterial development. The current results were consistent with the studies conducted by Moore^22^, Willems, et al.^9^, and Justé, et al.^7^, which revealed the optimal methods for storing thick juice below 30 °C.

According to the current results, the pH of the control tank dropped to 8.5 and 8.0, at the end of the storage period at 15 and 25 ºC. However, the application of biocides, hop-ß-acid and KEBOCID 310, kept the pH at approximately 9.0. On the other hand, pH declined to 7.32 at 35 ºC in the control tank, thus indicating the deterioration of the beet thick juice in this experiment. Using the biocides, hop-ß-acid and KEBOCID 310, could maintain the pH at 8.9 and 7.8, respectively. Lactic acid has been detected at 15, 25, and 35 ºC, according to our research resulgts, but only at relatively low concentrations of 50, 120, and 220 ppm, respectively. On the other hand, the use of the biocides hop-ß-acids, KEBOCID 310, and surface treatment with NaOH could hold the lactic acid content within the range of 50 to 80 ppm, demonstrating their beneficial role as preservatives for the thick beet juice. At the end of the storage period, it was determined that the concentrations of reducing sugars at 15, 25, and 35 ºC in control tanks were 0.17, 0.2, and 0.5%, respectively. By using the biocides, hop-ß-acid and KEBOCID 310, the reduction in reducing sugar level was reduced to a range between 0.01 and 0.02% at the three storage temperatures. The most obvious warning indications of deterioration was a large reduction in pH, usually to a low of 7.5 to 6.0, and a rise in reducing sugars at the same time. The pH reduction was most likely due to mesophilic bacteria converting sucrose to L-lactic acid, and this could account for the formation of reducing sugar. However, low pH generally did not correlate with large mesophilic bacterial populations.

Although the exact nature of this process is unknown. It is known that in highly alkaline conditions, sucrose undergoes a purely chemical reaction that results in lactic acid formation^45^. Since the primary source of lactic acid excretion—lactic acid bacteria—the existing data could provide some comformation to this theory. Despite the isolation and identification of bacteria that produce lactic acid—*B. cereus*, *B. subtilis*, and *B. licheniformis*—during this investigation, the current results revealed low concentrations of lactic acid and reducing sugars. This may be attributed to the slow microbial growth and metabolism at the specified Brix levels or the potent biocide activity, or both factors acting together.

Hein, et al.^25^ and Sargent, et al.^8^ proposed that an elevation in lactic acid is the explanation for the pH reduction during thick juice deterioration. However, Willems, et al.^9^ reported that they found no relationship between the deterioration of thick juice and any acid that was observed during storage. The outcomes weren’t always consistent^8^. Moreover, inadequate storage conditions, such as a low Brix index, high temperature, or possibly a vital oxygen concentration, might happen locally in a storage tank^46^, allowing species of bacteria to grow exponentially and result in thick juice deterioration. The majority of sugar companies consider this kind of deterioration to be a necessary expense of storing juice. Alternatively, the surface of the thick juice can be coated with a 25.0% sodium hydroxide solution to stop the formation of a microbial mat^13,25^.

This study examined the antibacterial effects of both biocides, hop-ß-acid and KEBOCID 310, at three different concentrations (20, 40, and 60 ppm) on the isolated species of bacteria. With an increase in biocide concentration, the effects of both biocides on the tested bacteria increased. In response to this concern, the application of biocides to the bulk of the juice before storage can impede the growth of microorganisms because of their antibacterial characteristics. Hop-β-acids are more preferable to the majority of sugar companies than formalin and dithiocarbamates due to their less hazardous and more natural alternatives^7,9,15^. Hop-β-acids have been demonstrated to display higher antibacterial action than α-acids^47^. Hop acids affect Gram-positive bacteria but have no effect on their endospores or on Gram-negative bacteria. Hop acids are believed to affect bacteria by disrupting the proper functioning of the membrane^48^, and by reducing the intracellular pH^49,50^.

At all concentrations (20, 40, 60 ppm) of Hop β-acids and at a concentration of 20 ppm of KEBOCID 310, *Bordetella muralis* and *Stenotrophomonas geniculata* were unaffected by the natural biocide, according to the experiment assessing the impact of biocides on the growth of the isolated strains of bacteria. There was little evidence linking these species of bacteria directly to contamination during sugar beet processing. Since these species of bacteria haven’t been identified as acid producers, we believe they play a less significant part in the degradation of the stored thick juice.

In keeping with the concept of a chemical-free refinery, natural biocides were being researched more and more for their antibacterial activity during thick juice storing^14^. These Hop components are considered harmless for humans and animals^16^. The results of this study demonstrated that, in contrast to 35 ºC, there had been no noticeable thick juice deterioration in the control tanks at 15 and 25 ºC, suggesting an insignificant effect of the biocides added to the thick juice bulk. This could indicate that the thick juice’s complex matrix quickly renders the active ingredient inactive.

The critical factors before and during the storage process of beet thick juice are the quality of the juice intended for storage, the conditioning process before storage by adjustment of pH and initial concentration (°Brix), storage temperature, type and concentration of the used disinfectant, as well as the sterilization of the storage tanks. The results confirmed the crucial role of temperature during thick juice storage, since storage at 15 and 25 °C clearly guaranteed a longer period of stability for the thick juice compared to 35 °C. Even though this storage temperature promotes microbial growth and increases the chance of degradation, it maintains the cost of storage low as well, particularly in countries with hot climates. In such an instance, optimizing the thick juice storage process requires the application of a biocide or physical defense combined with the high storage temperature. The type of biocide used is of particular importance, with a preference for a naturally derived one that has no harmful effects on human health compared to chemical disinfectants. The use of a natural biocide for preservation of thick juice is promising. It is also relevant due to the shorter processing period in sugar production from stored juice, which may allow residues of this biocide to remain in the final white sugar. As such, the natural biocide (Hop β-acid) is recommended in the preservation process at 35 °C and up to 120 days of storage. The addition of a hop extract significantly delayed thick juice degradation as measured by the stability in pH levels and the low microbial count. Additionally, the physical defense that uses NaOH for surface sealing and air removal can keep preservation costs low and also have no adverse health consequences.

## Materials and methods

### Biocides

Betastab^®^ XL (Hop ß-acids) was acquired from Beta Tec Hop Products Ltd (Malvern Hills Science Park; UK). KEBOCID 310 (sodium dimethyldithiocarbamate) was acquired from Keller & BohacekGmbH & Co. (Liliencronstraße 64, 40472 Düsseldorf, Germany).

### Layout of the pilot plant design

**Fig. 5:**
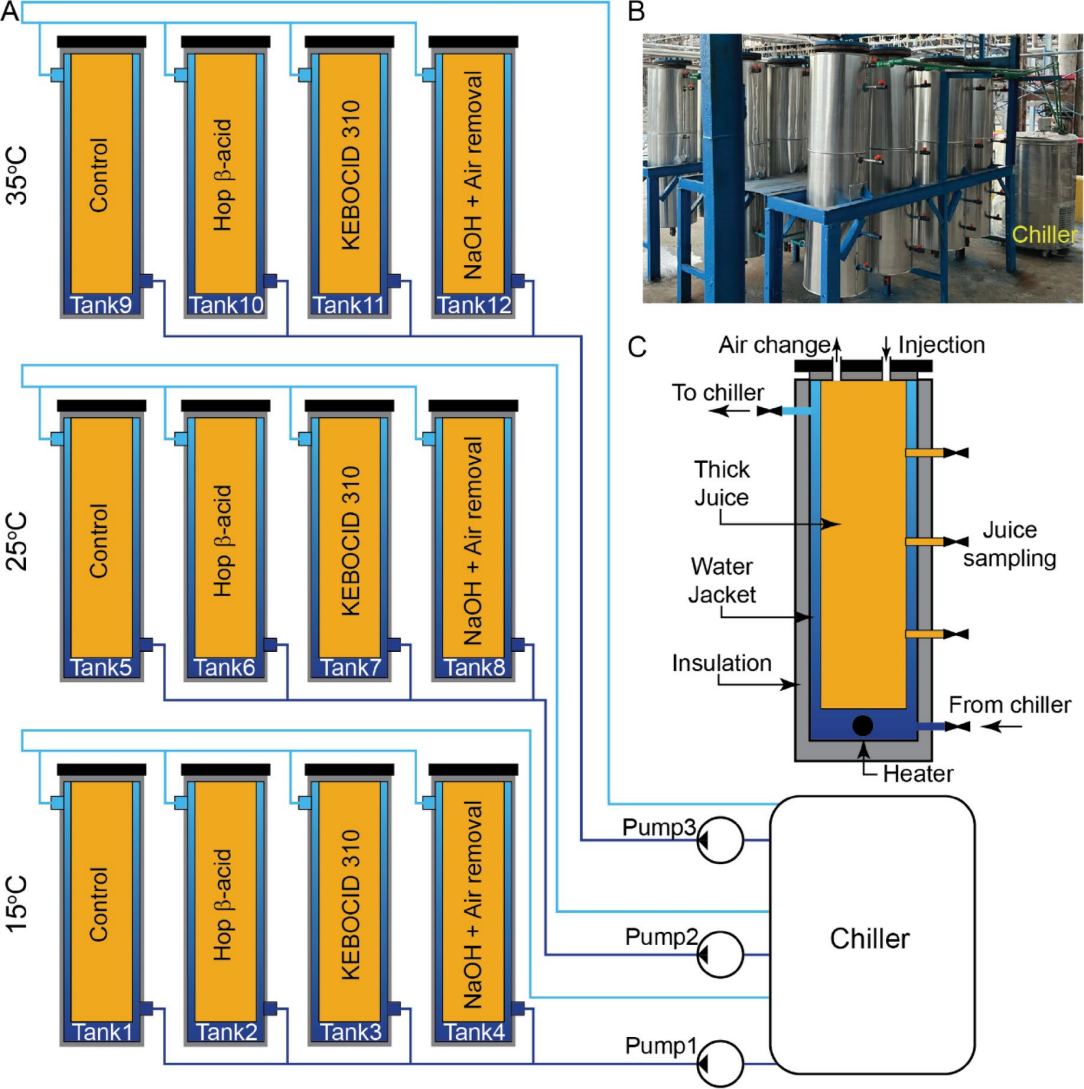
The pilot plant for storage of beet thick juice, including (**A**) Schematic diagram of the hydraulic and the control circuit of the pilot plant, (**B**) Site image of the 12 tanks stored at 15, 25, and 35 ℃, and (**C**) Details of a storage tank.

In order to simulate the storage conditions of a full-scale system, a small-scale pilot plant was constructed at the Alexandria Sugar Company in Egypt (Savola Foods, 30.819 N, 29.851 E). The pilot plant had a mechanism to regulate the temperature of the stored thick juice. To cool or heat the stored thick juice according to the altered setpoint, it relied on chilled water circulation and embedded heaters. The setup included twelve double-walled stainless steel tanks that could hold 80 L of beet thick juice, each with three sampling taps arranged vertically (Fig. 5). The tank could be heated or cooled by circulating water via a 50 mm thick jacket that encircled it. The exterior had 100 mm of rock wool insulation surrounding the water jacket and that eclosed the inner cylinder of the juice. An impeded temperature sensor monitored the tank temperature in real time and transmited the information to a differential controller for controlling the temperature. It sent on/off signals for the circulation pumps based on the difference between the tank temperature and the set points defined by users. During the cooling mode, the circulating water was kept cold using a chiller that was utilized to provide cooling for the whole pilot plant. Consequently, each group of tanks was cooled upon request by managing its corresponding circulation pump. During the heating mode, circulation pumps were deactivated, and the locally implanted heaters at the base of each water jacket were activated by the controller.

### Sampling and long-term storage conditions

Alexandria Sugar Company in Egypt provided the beet thick juice with 70 °Brix from the 2023 manufacturing season. The thick juice was collected in sterile 1,000-L polyethylene cubes following the evaporation stage, and the cubes were then kept at 4 °C until needed. When required, 25.0% NaOH and sterile demineralized water were used to adjust the pH of the thick juice and °Brix, respectively. Thick juice with °Brix values of 67, 68, and 69 was used for the storage experiment. In order to prevent sucrose nucleation, these circumstances kept the thick juice steady at a supersaturation coefficient of less than 1.0^6,20^. Examination of pH, lactic acid concentration, and reducing sugar content, all of which influence the durability of thick juice quality during storage, were determined corresponding to bacterial total count (CFU/mL). In addition to a control tank, three distinct treatments were utilized at each temperature group of 15, 25, and 35 °C (Table 4). In compliance with industry standards, a 10% aqueous alkaline solution of Betastab^®^ XL (Hop-ß-acids)^35^ as well as KEBOCID 310 (sodium dimethyldithiocarbamate)^9^ were applied to the thick juice at 40 ppm, as representatives of natural and chemical biocides, respectively^7,9,15,18,25^. The final treatment involved the physical removal of air using a blower and a microbiological filter affixed to one opening of the tank lid, in conjunction with the application of a 25% sodium hydroxide solution over the juice surface. Using official ICUMSA GS 2/3–10 methods^51^, biweekly estimates of pH, lactic acid concentration, and reducing sugar content, were made over the 180-day storage experiment. Samples of the stored thick juice were collected every 30 days of storage for determination of the bacterial diversity.

**Table 4:** Treatments of beet thick juice during 180 days of storage at 15, 25, and 35 ºC and ºbrix 67, 68, and 69 at an initial pH 9.2 using 40 Ppm of hop-ß-acid and KEBOCID 310 biocides, and surface sealing using 25.0% NaOH along with air removal.

Treatment temperature (ºC)	Tank No.	°Brix	Biocides (40 ppm)		Surface sealing
			hop-ß-acid	KEBOCID 310	25.0% NaOH and air removal
15	T1	67	–	–	–
	T2		X	–	–
	T3		–	X	–
	T4		–	–	X
25	T5	68	–	–	–
	T6		X	–	–
	T7		–	X	–
	T8		–	–	X
35	T9	69	–	–	–
	T10		X	–	–
	T11		–	X	–
	T12		–	–	X

### Microbiological analysis

#### Media used for isolation of bacteria

All media used for isolation of bacteria were acquired from OXOID (Oxoid Limited, Basingstoke, UK). De Man Rogosa Sharpe agar (MRSA/CM361) was used for isolation of bacteria. Columbia agar with 5–7% sheep blood (CAwSB/PB5029A) was used for isolation of fastidious bacteria (Table 5).

**Table 5:** Growth media and incubation conditions for monitoring of bacteria during long-term thick juice storage.

Microbiological parameter	Growth medium	Incubation temperature (°C)	Incubation time (day)	Incubation conditions
Lactic acid bacteria	MRSA/CM361	37	3	aerobic
Fastidious colony count	CAwSB/PB5029A	37	6	aerobic
Nutrient agar	–	37	3	aerobic

### Isolation of bacteria

Every 30 days of storage, samples of the stored thick juice were taken from each storage tank. All samples were collected in sterile glass bottles from the three taps along each storage tank, and the samples were promptly brought to the laboratory for bacterial isolation. Using the dilution plate method^52^, each of the 9 mm-diameter Petri dishes with the isolation media (Table 3) received 2.0 mL of the appropriate juice dilution. After that, the plates were incubated for three days at 37 °C under aerobic conditions. The plates containing the Columbia agar-sheep blood medium were incubated for six days prior to the growth of fastidious bacteria under aerobic conditions. In each sample, the number of developed colonies was counted together with the colony forming units (CFU/mL) in each mL of the juice. Nutrient agar^53^ was used to purify the resulting bacterial isolates at 37 °C.

### Molecular identification of the bacterial isolates

#### DNA isolation

The isolates of bacteria obtained in this study were separately grown in nutrient broth for 24 h at 37 ºC. After centrifugation (10,000 rpm at 4 ºC for 10 min), the bacterial cells were obtained in microfuge tubes. Eight hundred µL of CTAB buffer (3% CTAB, 1.4 M NaCl, 0.2% Mercaptoethanol, 20 mM EDTA, 100 mM TRIS-HCl pH 8.0 and 1.0% PVP-40) were added to each tube^54^. After incubation at 65 °C for 30 min, 800 µL of CI Mix with the composition of 24 mL chloroform and 1.0 mL isoamyl alcohol, were gently added and mixed with the tube contents. Clear supernatants were separately obtained by centrifugation (10,000 rpm at 4 ºC for 10 min). For DNA precipitation, a double volume of isopropanol (precooled at -20 °C) was added to each tube and gently mixed. The samples were incubated at 4 °C overnight, thereafter centrifugation (13,000 rpm at 4 ºC for 10 min). The supernatant was discarded and the pellets were separately pooled and washed with 200 µL washing buffer (76% ethanol and 10 mM ammonium acetate). The washing buffer was carefully decanted and the pellets were individually suspended in 200 µL TE buffer supplemented with 10 mg/mL RNase. After incubation at 37 °C for 30 min, 100 µL of 7.5 M ammonium acetate and 750 µL ethanol were added and mixed gently. Samples were centrifuged at 13,000 rpm for 10 min at room temperature. The supernatants were completely discarded and the pellets were separately suspended in 100 µL sterile distilled water.

### PCR and sequencing

The extracted DNA was then used as a template for PCR to amplify the 16 S rRNA gene. In the PCR tubes, 1.0 µL of DNA template, 1.0 µL of 2.5 mM dNTP mix, 0.2 unit of Taq polymerase, 5.0 µL of 10x complete buffer, 40.0 µL of sterile ddH_2_O, 10 pmol of 27 F (5′-AGA GTT TGA TCC TGG CTC AG-3′), and 10 pmol of 1492R (5′-GGT TAC CTT GTT ACG ACT T-3 ′) were used^55^. PCR amplification was carried out using the following sequence: one round of amplification consisting of denaturation at 95 °C for 15 min followed by 30 cycles of denaturation at 95 °C for 20 s, annealing at 50 °C for 40 s and extension at 72 °C for 1.0 min, with a final extension step of 72 °C for 5 min. The PCR products were then purified with the SolGent PCR Purification Kit-Ultra (SolGent, Daejeon, South Korea)^56^ prior to sequencing. The purified PCR products were confirmed on 1.0% agarose gel by electrophoresis using size marker. The amplified 16 S rRNA gene was sequenced in the forward and reverse directions using an ABI Big Dye Terminator (v 3.1) cycle sequencing kit (Applied Biosystems, Foster City, Cal., USA) and an ABI 373 0XL DNA analyzer (Applied Biosystems, Foster City, Cal., USA).

### Phylogenetic analysis

Contiguous sequence of each isolate of bacteria in this study was produced using the DNASTAR computer package (version 5.05). The most similar sequences to those in this study were downloaded from GenBank database (Nucleotide BLAST: Search nucleotide databases using a nucleotide query). All sequences in this analysis were aligned using MAFFT^57^ with the default options. Alignment gaps and parsimony uninformative characters were optimized by BMGE^58^. Maximum-likelihood (ML) and Maximum parsimony (MP) phylogenetic analyses were performed using MEGA X (version 10.2.6)^59^. The robustness of the most parsimonious trees was evaluated by 1000 bootstrap replications^60^. The best optimal model of nucleotide substitution for the ML analyses was determined using Akaike Information Criterion (AIC) as implemented in Modeltest 3.7 ^61^. The phylogenetic tree was visualized using MEGA X (version 10.2.6), and the resulting tree was edited and saved as TIF.

In vitro estimation of biocides activity.

The well diffusion method^62^ was employed for the determination of the inhibition zone of the two commercial biocides (hop-ß-acid and KEBOCID 310) on the tested strains of bacteria. Gradual concentrations (20, 40, and 60 ppm) were individually prepared from each biocide using distilled water. After the tested strains of bacteria were cultured for 24 h in nutrient broth medium, Petri plates were separately inoculated with spore suspensions (1.5 × 10^8^ spore/mL = 0.5 McFarland standard solution). The bacterial spore solutions were uniformly disseminated using sterile cotton swaps on Petri plate containing nutrient agar (NA). Each of the five wells (5 mm diameter) was separately supplemented with 50 µL of the biocides concentrations under test. The plates were then incubated aerobically for 24 h at 37 ± 1 °C. Following incubation, the suppression of bacterial growth was measured in mm. Three triplicates of each test were conducted.

### Impact of storage time on the quality parameters of the thick juice

The CFUs of each bacterial strain were calculated per one mL in every sample. pH was determined for the different thick juice samples using a Mettler Toledo Multiparameter (Model: Seven Excellence, USA). The concentration of lactic acid in each sample was measured by the direct determination with QuantiQuik™ L-Lactic Acid Quick Test Strips (QuantiQuik™; Catalog number QQLLAC10).

#### Determination of reducing sugars

The Berlin Institute Method was applied^20^ for the determination of reducing sugars. A mixture of 10.0 mL Muller’s solution and 0.5 g of thickened beet juice in 20.0 mL deionized water was prepared. After that, the mixture was placed in a boiling water bath. Once the mixture had cooled, 5.0 mL of 5.0 N acetic acid and an excess of 0.025 N iodine solution were added until a green tint appeared. Two drops of 1.0% starch indicator solution were then added, and the combination of the two was well mixed. After that, back titration was performed using a 0.025 N sodium thiosulfate solution until a light blue color appeared. In place of the sample, 20.0 mL of deionized water was used for the blank. The amount of reducing sugar was calculated according to the following formula:

$$\% \text{Reducing}\:\text{sugars}=\frac{{\text{mL}\:\text{Thiosulfate}}_{\text{Blank}}-{\text{mL}\:\text{Thiosulfate}}_{\text{sample}}-\:0.2}{\text{g}\:\text{Sample}}\:\times\:10$$

0.2 is the sucrose correction due to its reducing action.

### Statistical analysis

SPSS (Version 25) was utilized to conduct descriptive statistics, including means and standard errors. The data underwent analysis of variance (ANOVA: one-Factor with Replication), which was^63^. Following the ANOVA, post-hoc comparisons were conducted using the Tukey Honestly Significant Difference (Tukey HSD) test that was expressed as letters on the standard deviation.

## Conclusion

The present study had the objective of constructing a pilot plant to store beet thick juice for six months under Egyptian climate. Three matrix experiments at 15, 25, and 35 ºC were used to examine the stability of the thick juice during storage in relation to microbiological and chemical changes. A 10% aqueous alkaline solution of Betastab^®^ XL (Hop ß-acids), KEBOCID 310 (sodium dimethyldithiocarbamate), and 25.0% NaOH accompanied with air removal for surface sealing, were utilized as preservatives. The study examined the correlation between the microbial count, pH, reducing sugar content, and lactic acid concentration throughout the storage period. By the end of the storage period, the control tank at 35 ºC exhibited considerable degradation along with a drop in pH, while the remaining tanks displayed no degradation. Using 16 S rRNA sequencing, eight different species of bacteria species were isolated. These were *Bacillus cereus*, *B. licheniformis*, *B. paralicheniformis*, *B. subtilis*, *Bordetella muralis*, *Brevibacillus agri*, *Pseudomonas juntendi*, and *Stenotrophomonas geniculata*. The antibacterial efficacy of three biocide concentrations against the isolated species was evaluated. Both biocides’ effects on the bacteria under test increased with an increase in biocide concentration. Because microbial activity is one of the main problems with handling sugar solutions, this study was directed towards examining how this affects the quality of the stored thick juice. The primary cause of this activity is the presence of bacteria that either originated from the planting soils or the air. The following conclusions were drawn from the the data obtained in the present study. First, there was no observable rise in the microbial activity at the storage temperatures between 15 and 35 °C, and during storage durations of up to 120 days, especially when applying biocides or NaOH to keep the pH high. These practical settings comply with the standards of the sugar factories. A minor rise in bacterial growth was noted after 180 days of storage, but there is was discernible impact on the purity of the juice. After more than 150 days of storage, the juice began to degrade when the temperature reached 35 °C; however, this reaction was also inhibited by the use of biocides or NaOH. These findings demonstrated that the risk of storing thick juice with high Brix values was negligible if the storange time does not go beyond 150 days and 25 °C. While longer storage times and higher temperatures may have minor effects, it can be managed with the use of biocides and pH-adjusting reagents.

## Electronic supplementary material

Below is the link to the electronic supplementary material.

Supplementary Material 1

## Data Availability

“Data related to this manuscript is incorporated in the manuscript and supplementary data. The datasets generated and/or analyzed during the current study are available in the GenBank (https://blast.ncbi.nlm.nih.gov/Blast.cgi? PAGE_TYPE=BlastSearch; accession numbers PQ220143, PQ220147, PQ220148, PQ220154, PQ220155, PQ220156, PQ220157, PQ349481, PQ349482, PQ349613, PQ349730)”.
