# Supplementary material for: Bacterial biodiversity and optimization of pilot plant-based storage parameters of beet thick juice under Egyptian environmental conditions
Source: Sci Rep. 2025 May 16;15:17095. doi: 10.1038/s41598-025-99870-4 (PMC12084369; doi:10.1038/s41598-025-99870-4)
Supplement: Supplementary file 1 — Supplementary Material 1 [file 41598_2025_99870_MOESM1_ESM.docx]

**Bacterial biodiversity and optimization of a pilot plant-based storage parameters of beet thick juice under Egyptian environmental conditions**

Marwa Abdelhak^1^, Osama Abdel-Hafeez Mohamed Al-Bedak^2,3^, Mahmoud N. Abdelmoez^4,5^, Adel Ahmed Abdellah^6^, El-Sayed Abdel-Rahman^7^, Mohamed M. Abd El-Wahab^8,*^

^1^ Department of Science and Technology of Sugar Industry, Faculty of Sugar and Integrated Industries Technology, Assiut University, Assiut 71511, Egypt; (M.A.) [marwa_abdelhak@aun.edu.eg](mailto:marwa_abdelhak@aun.edu.eg) (0009-0000-3762-2792)

^2^ Assiut University Mycological Centre (AUMC), Assiut University, Assiut, 71511, Egypt; (O.A.M.A.-B.) [osamaalbedak@science.au.edu.eg](mailto:osamaalbedak@science.au.edu.eg) (0000-0003-0465-619X)

^3^ ERU Science & Innovation Center of Excellence, Egyptian Russian University, Badr city 11829, Cairo, Egypt

^4^ Department of Mechanical Power Engineering, Faculty of Engineering, Assiut University, Assiut 71511, Egypt; (M.N.A.) [mnady86@aun.edu.eg](mailto:mnady86@aun.edu.eg)

^5^ Institute for Life and Medical Sciences, Kyoto University, Japan.

^6^ Alexandria Sugar Company (Savola Foods), Burg El Arab 21934, Alexandria, Egypt; (A.A.A.) [adel.abdellah@savola.com](mailto:adel.abdellah@savola.com)

^7^ Department of Food Science and Technology, Faculty of Agriculture, Assiut University, Assiut 71511, Egypt; (E.A.) [e.soliman@aun.edu.eg](mailto:e.soliman@aun.edu.eg)

^8^ Department of Chemistry, Faculty of Science, Assiut University, Assiut, Egypt; (M.M.A.) [wahabm@aun.edu.eg](mailto:wahabm@aun.edu.eg)

Corresponding author: [wahabm@aun.edu.eg](mailto:wahabm@aun.edu.eg)

***
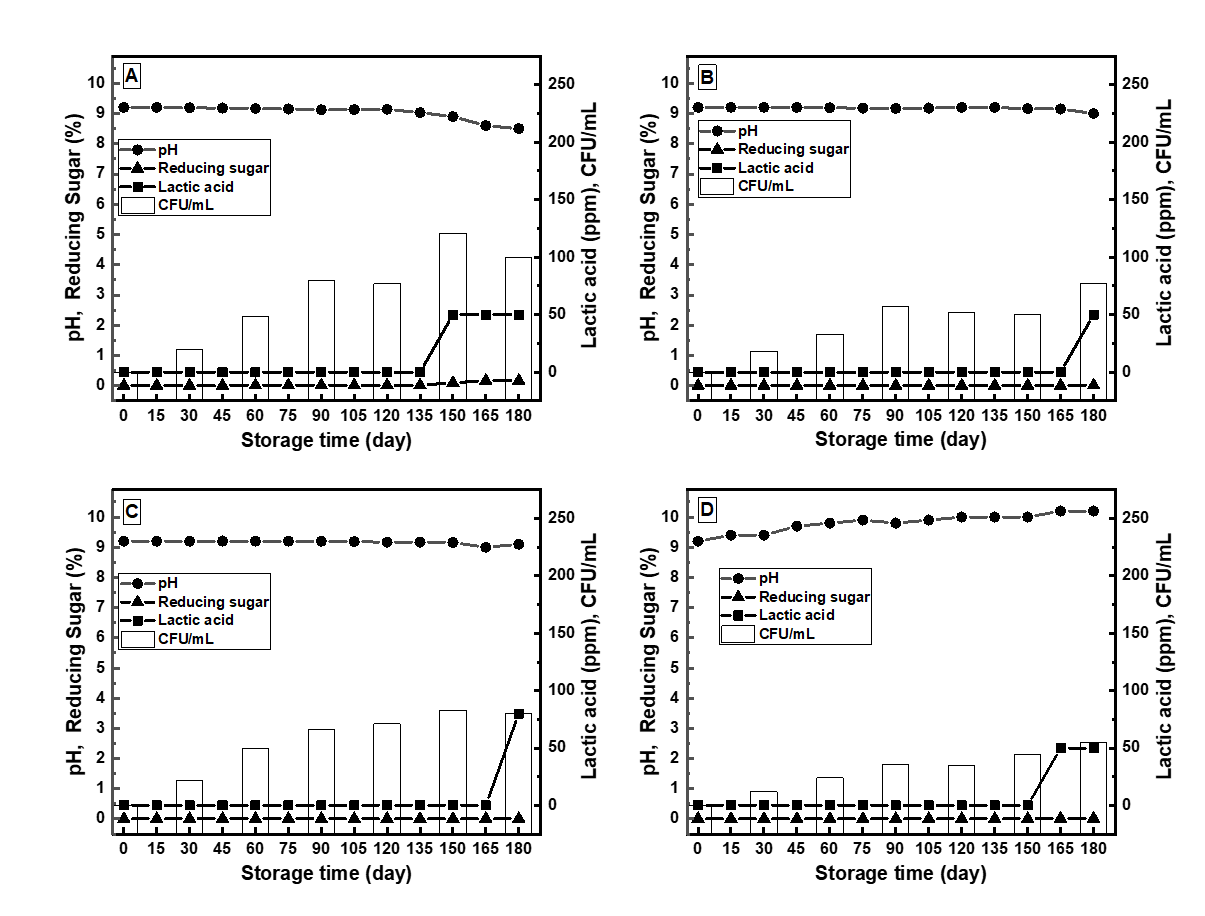
***

**Figure S1.** Effect of storage time (180 days) at 15 ºC on the microbial growth (CFU/mL) and the chemical parameters (pH, reducing sugars, and lactic acid) of beet thick juice. (A) Control tank (B) tank with Hop ß-acids (C) tank with KEBOCID 310 (D) tank with surface treatment.

**
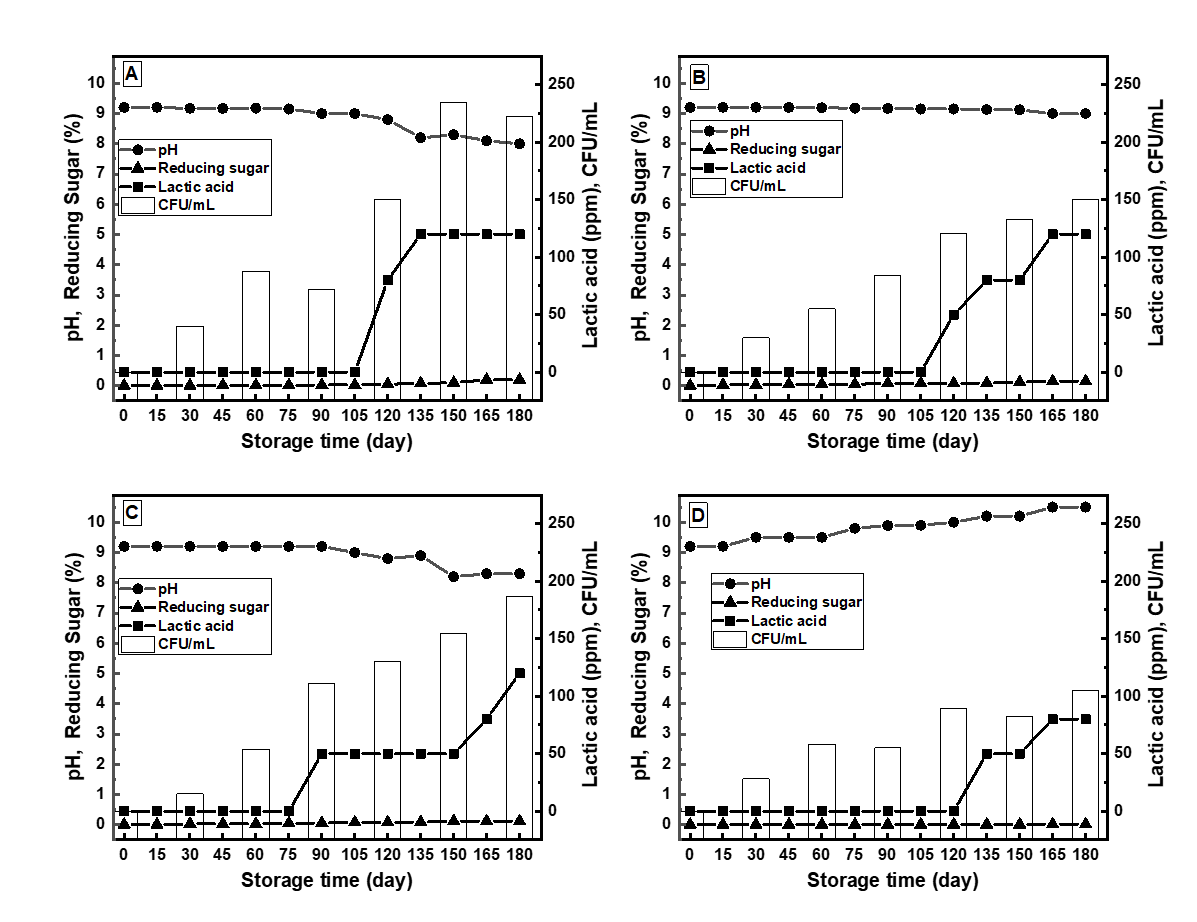
**

**Figure S2.** Effect of storage time (180 days) at 25 ºC on the microbial growth (CFU/mL) and the chemical parameters (pH, reducing sugars, and lactic acid) of beet thick juice. (A) Control tank (B) tank with hop-ß-acid(C) tank with KEBOCID 310 (D) tank with surface treatment.

**
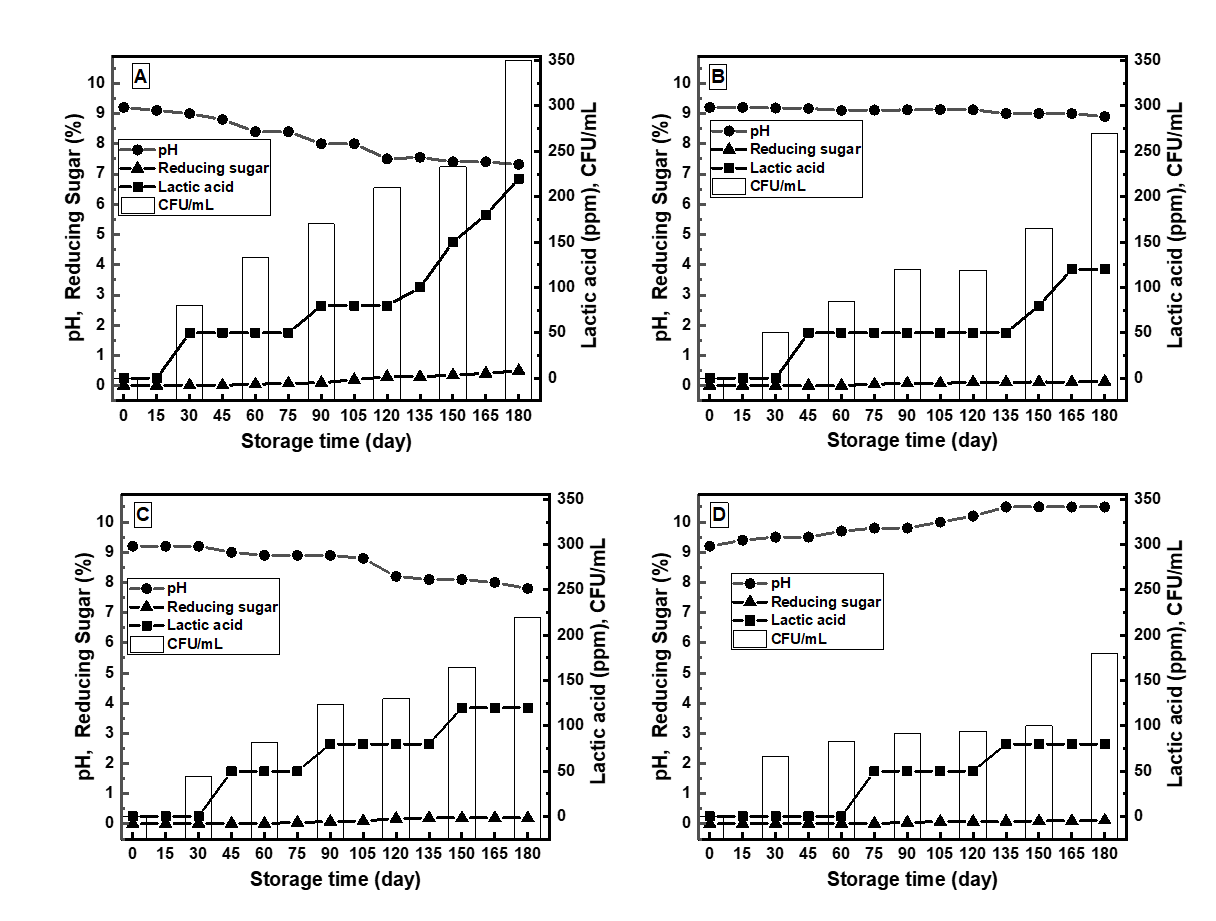
**

**Figure S3.** Effect of storage time (180 days) at 35 ºC on the microbial growth (CFU/mL) and the chemical parameters (pH, reducing sugars, and lactic acid) of beet thick juice. (A) Control tank (B) tank with hop-ß-acid(C) tank with KEBOCID 310 (D) tank with surface treatment.
